# Multi-Objective Optimization of Process Parameters during Micro-Milling of Nickel-Based Alloy Inconel 718 Using Taguchi-Grey Relation Integrated Approach

**DOI:** 10.3390/ma15238296

**Published:** 2022-11-22

**Authors:** Muhammad Sheheryar, Muhammad Ali Khan, Syed Husain Imran Jaffery, Mansoor Alruqi, Rehan Khan, M. Nasir Bashir, Jana Petru

**Affiliations:** 1 School of Mechanical and Manufacturing Engineering (SMME), National University of Sciences and Technology (NUST), Islamabad 44000, Pakistan; 2 Department of Mechanical Engineering, College of Electrical and Mechanical Engineering (CEME), National University of Sciences and Technology (NUST), Islamabad 44000, Pakistan; 3 Department of Mechanical Engineering, Shaqra University, Shaqra 11911, Saudi Arabia; 4 Department of Machining, Assembly and Engineering Metrology, Mechanical Engineering Faculty, VŠB-Technical University of Ostrava, 17. listopadu 2172/15, 708 00 Ostrava, Czech Republic

**Keywords:** Inconel 718, machining, multi-objective optimization, grey relational analysis

## Abstract

This research investigates the machinability of Inconel 718 under conventional machining speeds using three different tool coatings in comparison with uncoated tool during milling operation. Cutting speed, feed rate and depth of cut were selected as variable machining parameters to analyze output responses including surface roughness, burr formation and tool wear. It was found that uncoated and AlTiN coated tools resulted in lower tool wear than nACo and TiSiN coated tools. On the other hand, TiSiN coated tools resulted in highest surface roughness and burr formation. Among the three machining parameters, feed was identified as the most influential parameter affecting burr formation. Grey relational analysis identified the most optimal experimental run with a speed of 14 m/min, feed of 1 μm/tooth, and depth of cut of 70 μm using an AlTiN coated tool. ANOVA of the regression model identified the tool coating parameter as most effective, with a contribution ratio of 41.64%, whereas cutting speed and depth of cut were found to have contribution ratios of 18.82% and 8.10%, respectively. Experimental run at response surface optimized conditions resulted in reduced surface roughness and tool wear by 18% and 20%, respectively.

## 1. Introduction

High-speed micro-machining is being used by industries extensively as it removes metal more quickly than traditional machining. Nevertheless, it poses issues since it generates more heat and requires more frequent chip removal from the cutting zone [1]. In particular, micro-machining of super alloys presents a major challenge for the production of high-precision miniature products having micro features [2]. Production techniques used in various industries are expected to become productive and sustainable with the constant evolution of technology. In the aviation industry, particularly, aircraft engines, for example, are being improved in efficiency to save money on fuel costs [3,4]. Blades, discs, and other components of aircraft engines have been made with Inconel 718 due to its superior mechanical properties including strength, corrosion resistance, and high temperature creep resistance [5]. Nickel, chromium, iron, and other constituents make Inconel 718 resistant to wear and corrosion [6,7]. Inconel 718’s low machinability requires deeper investigation of the manufacturing process. Resultantly, it is used in the manufacture of various rocket and gas turbine parts. Electrochemical micro-machining has been optimized for Inconel 718 using a variety of techniques. Although drilling operation is used for Inconel 718 using electrochemical machining techniques, micro-machining Inconel 718 still poses problem [8,9,10]. The miniaturization of industrial components with a wide range of functions and acceptable dimensional accuracy is a widely researched topic. The mass production of small parts and components is possible through the use of micro-machining technology. Small and precise 3D objects, ranging in size from 1 nm to 0.99 nm, are created using different material removal processes [11,12,13]. Recent years have seen a significant increase in the demand for micro parts and components of all kinds including inkjet printing heads and pharmaceutical micro pump delivery systems. The manufacture of miniature parts demands the use of more precise tooling and processes that are required to be dependable and repeatable. Many researchers have analyzed different ways to manufacture micro-components, including laser manufacturing, ultrasonic photolithography, and ion beam machining [14,15,16,17]. Inconel alloys are one of the most thoroughly investigated materials in the published literature because of their utility in a vast range of industries [18,19].

In order to increase the machining performance of Inconel 718, various arrangements including different coatings, coolants, machining settings, and laser-assisted machining for preheating the workpiece are adopted [20,21,22]. During the machining process, specific cutting energy increases in comparison to traditional machining because of the high temperature strength. Tool wear and surface roughness were studied by Irfan et al. [23] using 48 m/s high-speed micro-machining on Inconel 718. Diamond-like coatings (DLC) and TiAlN + WC/C coatings performed well in terms of tool wear and the development of built-up edges (BUE). Another observation was that compared to tools coated with AlTiN and TiAlN + WC/C, the DLC-coated tool produced the lowest surface roughness. The literature also highlights significant research works related to parametric optimization. One such study considered current, voltage, and gas flow rate during regulated metal deposition welding using ASTM A387 grade 11 steel [24]. The satisfaction function approach was implemented using Taguchi DOE with output responses of depth of penetration and heat affected zone. Input parameters of 100 A current with 16 V voltage and 21 L/min gas flow rate were identified and validated as optimal. Similarly, another noteworthy work considered bead height and bead width in addition to depth of penetration and heat affected zone as preferred output responses during gas metal arc welding for low alloy steel [25]. Optimal settings were identified with 92 A current, 13 V voltage, and 21 liter/min gas flow rate. In addition, a substantial work related to the comparative analysis of different joining techniques for NiTi shape-memory alloys is also available in the literature [26]. It includes their main characteristics, benefits, limitations, and applications. Analysis of surface roughness by Lu et al. [27] concluded that machining parameters are interrelated in terms of their collective effect and so the optimum machining parameters were worked out using multi objective optimization. During the micro-milling of Ti6Al4V alloy, Aslantas et al. [28] observed that tool coatings affected cutting force, tool wear, and machining quality. As tool wear increases, cutting force rises, resulting in reduced machining accuracy as indicated by elevated surface roughness. According to Ozel et al. [29], during micro-milling Ti6Al4V, the cBN-coated tool outperformed the uncoated one in terms of machining and wear quality. Different tool coatings were explored by Aramcharoen et al. [30] with micro-milling hardened tool steel. In terms of edge chipping and flank wear, coatings such as TiN were found to be superior to TiAlN, although TiAlN developed more burr width in comparison with uncoated tools. It was shown that cutting speed had a greater effect on surface roughness than depth of cut or feed rate. According to Rahman et al., MQL is more consistent and stable than dry machining in the micro-machining of Inconel 718. Wear on tools from dry-cut operations is substantially greater than from wet-cut operations due to the slow dissipation of generated heat at the tool/workpiece interface [31,32]. Some input parameters have been explored by researchers to increase the quality of the machined surface during micro-machining. In one such study, Attanasio et al. [33] focused on the impact of the microstructure (burr generation, tool wear, and cutting forces) on the quality of machining (burr production). Similarly, surface uniformity was examined by Zhanwen Sun and Suet [34] to improve machining quality. Different input factors such as feed rate and spindle speed were analyzed. In another related study, burr formation and surface roughness were found to reduce by altering spindle speed tilt angle [35]. Multi-objective optimization is an effective practice used by many researchers for the collective optimization of manufacturing systems in terms of productivity and sustainability [36]. Joshi et al. [37] utilized multi-objective optimization to generate Pareto optimal solutions for micro-turning and micro-milling applications. NSGA-II, MOALO, and MODA generated Pareto solutions were then compared using a complex proportional assessment (COPRAS). Tien et al. [38] employed a multi-objective particle swarm optimization technique using the output response of tool life, surface roughness and power consumption during the high speed milling process. Tool wear and surface roughness were improved by 9.87% and 5.95%, respectively, whereas power consumption was improved by 10.49% by careful selection of identified input parameters. 

According to the available literature, several researchers have used a variety of tool coatings in order to extend tool life and ensure compatibility with a wide range of materials during micro-milling procedures. In most instances, tool wear was not taken into consideration in the research, hence the effect of different tool coatings on machining quality is unexplored and presents a research gap. In addition, owing to the reduced tool vibration and burr formation at higher cutting speeds, earlier research concentrated on high cutting speeds, whereas few studies examining the quality of micro machined components at lower cutting speeds are present, which forms another significant research goal. Consequently, this work attempts to fill the identified literature gaps and investigate the effects of different machining parameters on surface roughness, burr generation, and tool wear. 

## 2. Experimental Methodology

The various machining parameters such as cutting speed, depth of cut, and feed rate, were taken into consideration during design of experimental arrangement. The following sections address several aspects of experimental methodology.

### 2.1. Experimental Setup

**Figure 1:**
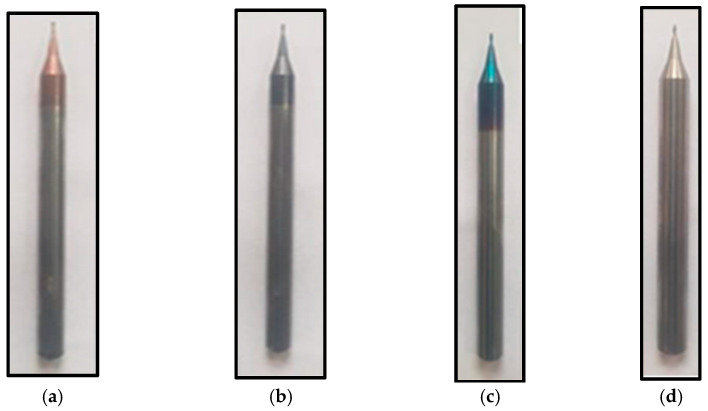
Different tools used in the current study (**a**) TiSiN, (**b**) AlTiN, (**c**) nACo, (**d**) un-coated.

CNC milling machine (PARPAS PHS-680, OMV, PARPAS, Italy) was used to perform micro-milling tests on nickel-based superalloy Inconel 718. Initially, a carbide end mill with a 12 mm diameter was used to level the work piece surface. Afterwards, the surface was employed as a point of reference for the design process. A tool pre-setter was used to ensure accurate z-axis measurements. The experimental parameters are listed in Table 1. Wedge-shaped cutting tools (tungsten carbide steel with 0.06-inch diameter) were used for experimentation. Different cutting tools used in this work are shown in Figure 1. Microtools with nACo, AlTiN, and TiSiN coated cutting edges had an average cutting-edge radius of 1.3 µm, 1.21 µm, and 3.0 µm, respectively. 

**Figure 2:**
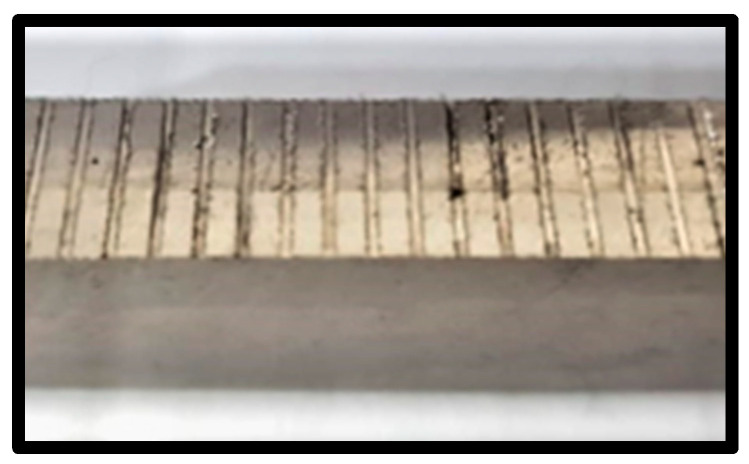
Milled workpiece having slot spacing of 2 mm.

The dimensions of the work piece, 146 mm × 10 mm × 22 mm, were prepared using EDM (KNUTH, Hamburg, Germany). Experiments were conducted with a 10 mm slot in the cutting length to reduce tool wear. Figure 2 shows the slot spacing, kept at 2 mm. The first step was to grind and polish the material. Kalling’s waterless itching agent was used for about 5 s before being washed away with water. Using a digital microscope (Olympus DXS1000, Olympus Corporation, Tokyo, Japan) and the ASTM standard method, the average grain size was determined to be 23.4 µm. The Vickers hardness of Inconel 718 was found at 361 HV using a Vickers Micro hardness tester (HAIDI, Dongguan, China). 

### 2.2. Design of Experiment 

Tool coatings (t_c), cutting speed (Vc), depth of cut (ap), and feed rate (fz) were chosen as input parameters as they have significant effects on surface roughness, wear rate, and burr formation [39,40,41,42]. Analysis of burr development, tool wear and surface roughness/finish were carried out using input variables. The range of levels of these parameters was based on the literature [43,44]. Table 2 presents the selected machining parameters and their levels. Main effect plots and ANOVA, based on Taguchi design of experiment [45], were used to analyze the contribution rations of input variable on output responses surface roughness, burr formation, and tool wear. All sixteen of these tests were repeated twice to ensure repeatability. 

### 2.3. Measurement of Responses

Burrs can form in a variety of sites, including the top, bottom, entrance, and exit burrs. In the current work, top and bottom burr height and width, were measured using a digital microscope (DXS-1000, OLYMPUS, Tokyo, Japan) at different magnifications based on the burr. The digital microscope (Olympus DXS1000) was used to determine the surface roughness of all slots as it enables the determination of micro-surface roughness in micro-milling operations. At the beginning of the machined slots, surface roughness was measured to check if tool wear had an impact on the result. ISO 4287 is the followed standard for measuring surface roughness. Moreover, the third response, i.e., tool wear, was also measured using the digital microscope (Olympus DXS1000). 

## 3. Results and Analysis 

The results achieved from the experiments for burr width, burr height, surface roughness, and tool wear are displayed in Table 3. There were multiple runs of each experiment, and the average of those runs was worked out as shown in the table. Effect of every input on output was then independently analyzed.

### 3.1. Effect on Tool Wear 

**Figure 3:**
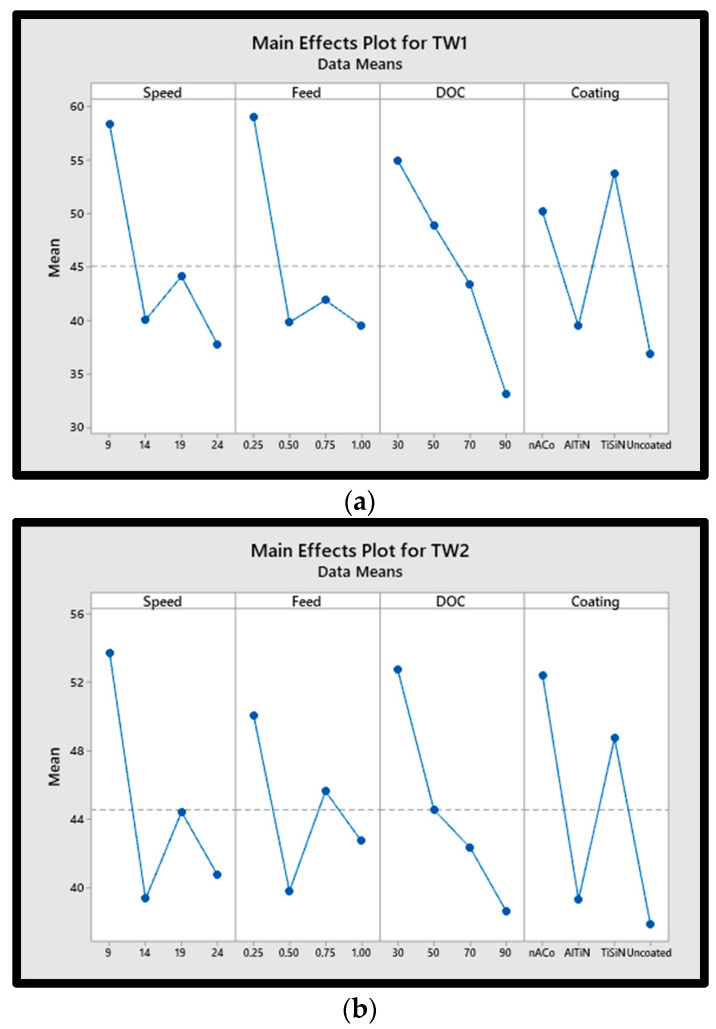
Main effects plot of tool wear (**a**) flute 1, (**b**) flute 2.

The finished product quality and the accuracy of the machining process are both adversely affected by tool wear [46], which is an irreversible process. The tool wear rate is directly proportional to the high temperature strength of the workpiece material [47,48], among other factors. Main effect plots for tool wear are shown in Figure 3. Here the individual effect of each input parameter is analyzed on tool wear progression. It is observed that all machining parameters including cutting speed, feed rate, DOC, and coatings have significant effects on tool wear. As seen in Figure 3, higher cutting speeds and moderate feed rates can reduce abrasive wear in the beginning. As a result of irreversible wear on tools, higher temperatures in the cutting zone can cause volumetric gain, which can lead to the workpiece material adhering to the tool’s cutting face, reducing the tool’s hardness, and increasing its wear rate. The hardness of the workpiece and the machining parameters used during the machining process affect the effective tool life of a cutting tool. Non-uniform abrasion of the active cutting edge, tool cutting face, and tool flank are responsible for the high tool wear rates. 

### 3.2. Effect on Surface Roughness

**Figure 4:**
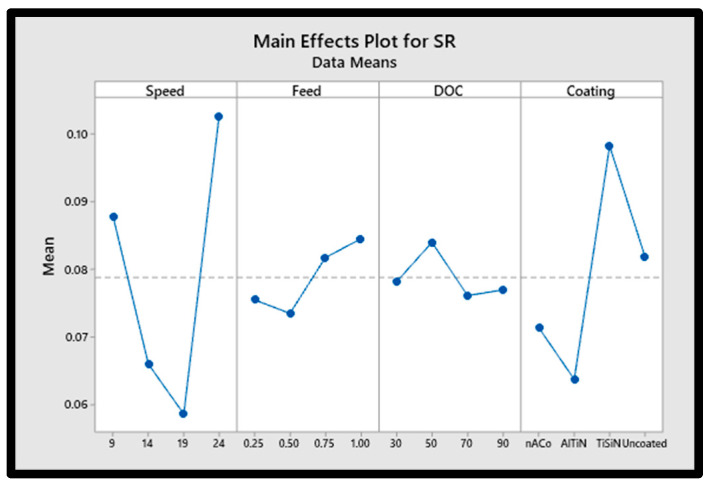
Surface roughness as a function of tool coating and cutting parameter.

Machined work piece surface roughness is affected by factors such as cutting-edge radius and tool coating, as well as cutting speed and depth-of-cut. The main effect plot of surface roughness based on input parameters is depicted in Figure 4. Inconel 718 micro-machining with a 10 mm cutting length yielded the lowest surface roughness values when using AlTiN coated tools, according to the main effect plot. An increase in cutting temperature may have been induced by an increase in the coefficient of friction. As a result of a greater cutting temperature and a lower feed/tooth radius, most of the material removal occurs through chip deformation. While surface roughness is reduced without grooves, friction between tool and workpiece increases burr development and facilitates chip deformation. As a result, cutting at a greater velocity with an AlTiN coated tool yielded the lowest surface roughness. Compared to AlTiN-coated tools, nACo-coated tools demonstrated the second lowest results for surface roughness. Surface roughness values were observed to be higher in TiSiN coated tools. Surface roughness values rise as a result of the increased cutting force and tool vibration [27]. The literature highlights that excellent surface quality can be produced with a minimal chip thickness with increasing cutting force [49]. Workpiece velocity relative to the cutting tool is referred to as feed rate. Feed per tooth is directly proportional to the feeding rate. Feed/tooth is the amount of material that each tooth of the cutting tool is capable of cutting. Because the cross-sectional area of the chip was expanding, the cutting load in the machining process was also increasing. When the cutting process is disrupted as a result of tool wear, it has a negative impact on the surface finish. The rate of tool wear increases as feed/tooth is increased. Increasing the feed rate from 0.5 to 0.1 µm/tooth resulted in increased surface roughness as demonstrated in Figure 4. 

In terms of surface finish, the tool’s cutting-edge radius is one of the most critical factors [50]. As cutting speed increases, the temperature rises, which in turn affects the roughness of the surface [51]. Research shows that the DOC has no significant impact on surface roughness. Surface roughness was shown to be more attributable to an enhanced ploughing effect at very small depths of cut, but as the DOC increased, the ploughing impact decreased and appropriate cutting occurred, resulting in a decrease in surface roughness. According to the literature, the surface quality deteriorated due to an increase in cutting force and vibration. 

### 3.3. Effect on Burr Formation 

**Figure 5:**
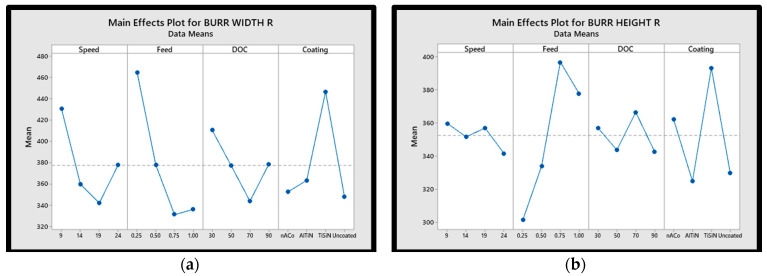

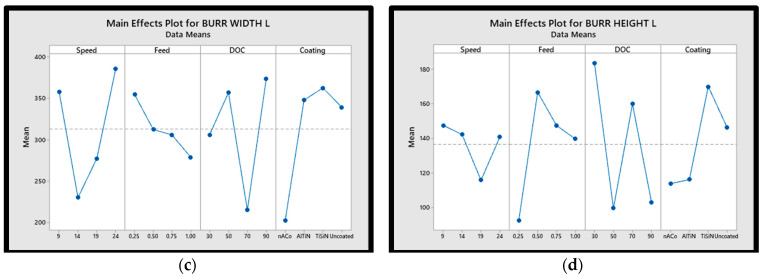
Main effects plots of (**a**) burr formation width down milling, (**b**) burr formation height down milling, (**c**) burr formation width up milling, (**d**) burr formation height up milling.

The burr width and burr height for both up milling and down milling were used as response variables in the current research. Main effect plots for burr width and burr height for both up milling and down milling are shown in Figure 5. The results indicated that the down milling operation produced the majority of the burr generated during the experiment. During the burr analysis, researchers focused their attention on the very top burr. Each slot’s maximum burr width and height were determined using a digital microscope. 

**Figure 6:**
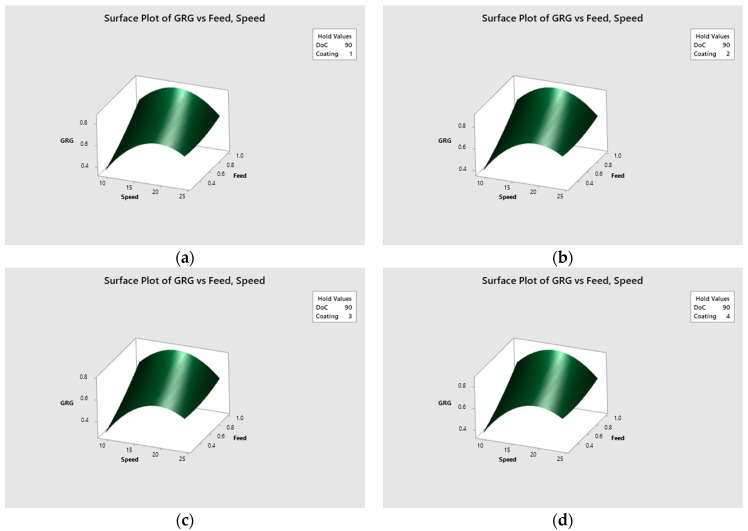
Surface plots of *GRG* vs tool coatings (**a**) nACo, (**b**) AlTiN, (**c**) TiSiN, (**d**) uncoated.

For the micro-machining of Inconel 718, burr is most likely created when the cutting length is set at 10 mm, as shown in the main effects plot. According to Figure 6, it was found that the burr width reduced with increasing cut depth when micro-machining Inconel 718. Uncut chips can be easily chipped off since burr is an uncut form of the chip. This makes it easier to chip off the worked piece at a higher depth of cut than at a lower depth of cut, which reduces burr formation. The tool with TiSiN-coating had a higher coefficient of friction, which aided to distort the material as the temperature rose, resulting in more burr development [52]. As the feed rate increased, it was observed that the burr width first increased, and then decreased. It was concluded that burr width reduced with a rise in the feed-to-cutting-edge radius. Additionally, it was determined that increasing the cutting speed led to a larger burr as various cutting speeds lead to considerable variations in cutting temperature. A broader burr is produced by machining at a higher speed because the workpiece deforms owing to higher cutting zone temperatures. The tool with TiSiN-coating had a higher coefficient of friction, which aided to distort the material as the temperature rose, resulting in more burr development. As the feed rate increased, it was discovered that the burr width first grew, and then decreased. It was also observed that burr width reduced with a rise in the feed-to-cutting-edge radius.

### 3.4. Optimization of Individual Process Responses

In the current investigation, the smaller is better model is adopted for burr formation, surface roughness, and tool wear. As inferred from the main effects plot, described in Section 3.3, output responses are optimized at varying conditions of input parameters. In order to validate the experimental design, confirmatory tests were then carried out for best and worst responses using identified input conditions. The results for output responses along with the input parameters are given in Table 4. The achieved results are confirming the reasonability of the experimental procedure.

### 3.5. Need for Multi-Objective Optimization

Table 4 analysis reveals that individual output responses optimize at different input variable levels. Due to this particular situation, it is necessary to conduct multi-objective optimization to collectively optimize the manufacturing output [53,54,55,56]. 

## 4. Multi-Objective Optimization Using Grey Relational Analysis

The research objective of achieving the optimum manufacturing output can be achieved with multi-objective optimization. Deng Julong [57] developed the methodology employed in this study in 1989. Deng Julong introduced the idea of the grey system for the first time in 1981, defining it as what is not explicitly expressed in black or white, therefore being grey. The goal was to process the data in a way that enables decision-making. Wang Ting [58] first proposed the grey relational grade in 1985. Grey relational analysis was carried out for multi-objective optimization in this study. By GRA the combined effects of input parameters on output responses can be examined and their combined integral contribution to each of the output responses can also be measured. Using their combined weightage, each set of input parameters can then be ranked accordingly. There are several steps [59], each of which is detailed below.

### 4.1. Pre-Processing Measured Data

This step involves converting each response value to a scale with extremes at 0 and 1. Using Equation (1), surface roughness, tool wear, and burr development are normalized because they are based on the smaller the better model.

(1) $$Z_{ij}=\frac{max\;\left(y_{ij},\;i=1,2,\ldots n\right)\;-y_{ij}}{max\;\left(y_{ij},i=1,2,\ldots n\right)\;-min\;\left(y_{ij},i=1,2,\ldots n\right)}$$

Here, *i* is equal to 1, 2, …, *n* and *j* is equal to 1, 2, …, *m*, where *m* is the total number of responses analyzed and index *n* is the total number of experimental data parameters.

### 4.2. Grey Relational Coefficient (GRC) Calculation

The grey relational coefficient (GRC) is then determined using Equation (2), with the processed data.

(2) $$\gamma \;\left(Z_{o},\;\right.Z_{ij})=\frac{\Delta min+\xi \Delta max}{\Delta_{oj}\left(k\right)+\xi \Delta max}$$

In this case, the value of *(Z_o_,Z_ij_)* is more than 0 and equal to or less than 1. *Z_ij_(k)* and *Z_o_(k),* where *Z_o_(k)* = 1 and *k* = 1, …, *m*, respectively, are the comparability and reference sequences. Additionally, deviation sequence is calculated using Equation (3).

(3) $$\Delta_{oj}\left(k\right)=|Z_{o}\left(k\right)-Z_{ij}\left(k\right)|$$

The values of Δ*min* and Δ*max* are equivalent to the least and biggest values of Δ*_oj_* (*k*). The distinguishing coefficient “ξ” is maintained at 0.5 if all parameters have equal weight. Usually, ξϵ∣0, 1∣.

### 4.3. Grey Relational Grade (GRG) Calculation

In the third step, the formulated GRCs are combined into a single grey relational grade (*GRG*). *GRG* is calculated using Equation (4), where *ω_r_* is the weight of the *r*th objective, whose total value is equal to 1 as shown by Equation (5). Manufacturers use client requirements or established policies to determine weight given to individual GRCs. In the current study, all responses are given equal weightage [47]. The obtained *GRG* can be maximized for optimum collective manufacturing output.

(4) $$\mathrm{Grade}\;\left(Z_{o},\;Z_{ij}\right)=\overset{n}{\underset{r=1}{\sum }}\omega_{r}\gamma \left(Z_{o},Z_{ij}\right)$$

(5) $$\overset{n}{\underset{r=1}{\sum }}\omega_{r}=1$$

### 4.4. GRG Rank

All the experimental runs were then marked with their *GRG* values, ranked from 1 to 16. The best run in the present set of experiments identified by the highest *GRG* value, and it is ranked first. Table 5 displays the experimental runs against their *GRG* values. With input parameters of cutting speed 14 m/min, feed rate 1 µm/tooth, depth of cut 70 µm, and AlTiN tool coating, experiment #8 yielded the highest *GRG* value.

## 5. Regression Analysis 

Regression modeling and its optimization was also carried out for elaborate machinability analysis. Afterwards, ANOVA was used to identify vital contributing factors and validation tests were conducted.

### 5.1. Regression Modeling of Multi-Objective Function

**Figure 7:**
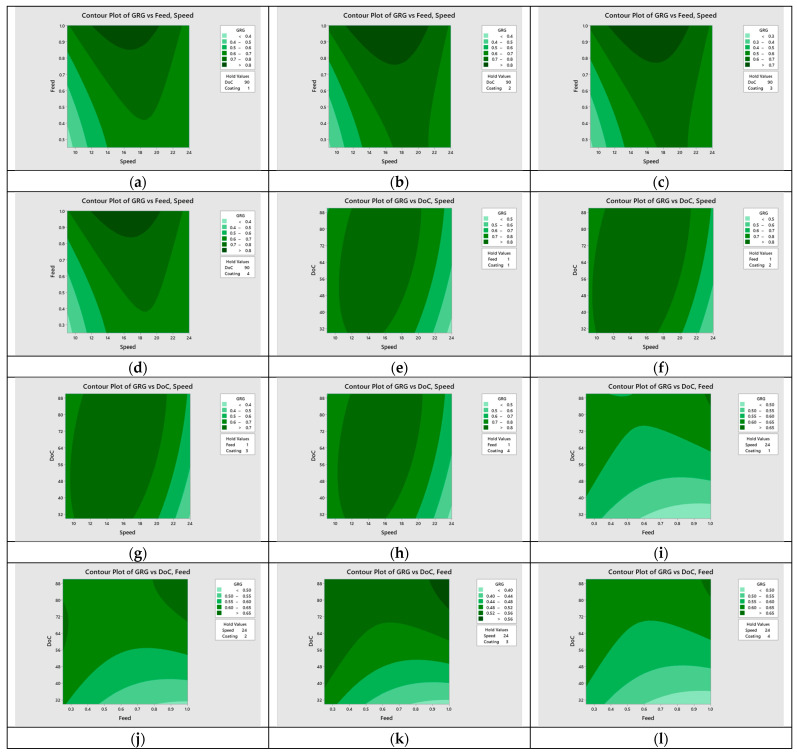
Contour plots of *GRG* vs. machining parameters (**a**) speed vs. feed, nACo; (**b**) speed vs. feed, AlTiN; (**c**) speed vs. feed, TiSiN; (**d**) speed vs. feed, uncoated; (**e**) speed vs. depth of cut, nACo; (**f**) speed vs. depth of cut, AlTiN; (**g**) speed vs. depth of cut, TiSiN; (**h**) speed vs. depth of cut, uncoated; (**i**) feed vs. depth of cut, nACo; (**j**) feed vs. depth of cut, AlTiN; (**k**) feed vs. depth of cut, TiSiN; (**l**) feed vs. depth of cut, uncoated.

Multi objective functions were made for the four discrete input parameters, i.e., tool coatings, as given by Equations (6)–(9). Since tool coating is a non-continuous categorical factor with four distinct levels: nACo, AlTiN, TiSiN, and uncoated. These four equations are valid for all input parameter values of the ranges selected in this study. Then RSM was used to carry out optimization of the regression models. The surface plots of *GRG* at various machining parameters are shown in Figure 6. The contour plots of *GRG* for all four tools are shown in Figure 7 at various machining parameters.

(6) $$\begin{array}{l} GRG\;\left(f,V,d,\;nACo\right)=-0.080+0.1056V+0.096f-0.00277d-\\ 0.00326V^{2}+0.202f^{2}-0.000036d^{2}-0.0253Vf+0.000279Vd+0.00362fd \end{array}$$

(7) $$\begin{array}{ll} GRG\;(f,V,d,\;AlTiN) & \\ = & -0.051+0.1056V+0.096f-0.00277d-0.00326V^{2}\\ + & 0.202f^{2}-0.000036d^{2}-0.0253Vf+0.000279Vd\\ + & 0.00362fd \end{array}$$

(8) $$\begin{array}{ll} GRG\;(f,V,d,\;TiSiN) & \\ = & -0.154+0.1056V+0.096f-0.00277d-0.00326V^{2}\\ + & 0.202f^{2}-0.000036d^{2}-0.0253Vf+0.000279Vd\\ + & 0.00362fd \end{array}$$

(9) $$\begin{array}{ll} GRG\;(f,V,d,\;Uncoated) & \\ = & -0.075+0.1056V+0.096f-0.00277d-0.00326V^{2}\\ + & 0.202f^{2}-0.000036d^{2}-0.0253Vf+0.000279Vd\\ + & 0.00362fd \end{array}$$

### 5.2. Analysis of Variance (ANOVA)

The ANOVA of regression model is given in Table 6. The contribution ratio of tool coating is found to be the highest at 41.64%, whereas the contribution ratio of speed is 18.82%. Contributions ratios from depth of cut and feed are 8.10% and 1.93%, respectively. With a 14.96% contribution ratio, speed was the most influential input parameter in square terms.

### 5.3. Regression Model Optimization

**Figure 8:**
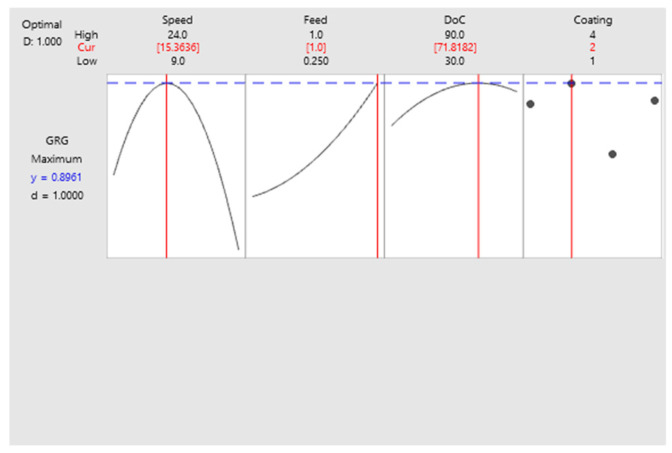
Response surface optimization of *GRG*. * 1 = nACo, 2 = AlTiN, 4 = uncoated.

To obtain the optimized machining parameter combination for the best output response, response surface optimization was conducted. Figure 8 displays the set of input parameters for optimized output. Additionally, the validation of results was obtained using additional experimentations.

### 5.4. Validation Experiments

Table 7 lists the machining parameters that were RSM-optimized together with the best run condition in the initial trials (experiment #8). Results from the validation of these circumstances showed significant improvement in all output responses. It was found that burr height (up milling) improved by 13.34%, burr height (down milling) by 10.58%, burr width (up milling) by 11.16%, burr width (down milling) by 9.81%, surface roughness by 18%, tool wear (flute 1) by 20.12%, and tool wear (flute 2) by 20.86%.

## 6. Conclusions

In the current investigation, machinability of Inconel 718 was assessed during micro-milling using uncoated and coated 0.5 mm diameter end mills. Input machining parameters were varied to analyze their effects on output responses including tool wear, surface roughness, and burr formation. MOO was conducted for overall improvement of system response. The following conclusions were reached during the conduct of research:

1. Selected input parameters were found to have significant effects on output responses as indicated by their main effect plots. Uncoated and AlTiN coated tools resulted in lower tool wear than nACo and TiSiN coated tools. In terms of surface roughness, AlTiN coated tools produced the least surface roughness whereas TiSiN yielded the highest surface roughness.
2. TiSiN coated tools resulted in the highest burr formation among all the coated and uncoated tools. Among other factors, feed was identified as the most influential parameter affecting burr formation.
3. The combination of input parameters for best and worst responses were found to vary substantially for each output response as evidenced from the identified machining conditions. This underlined the need for MOO for enhancing system productivity.
4. Grey relational analysis identified the most optimal experimental run with a speed of 14 m/min, feed of 1 µm/tooth, and depth of cut of 70 µm using AlTiN coated tools. Similarly, an experimental run at a speed of 24 m/min, feed of 1 µm/tooth, and a depth of cut of 30 µm using TiSiN coated tools was marked as the least optimal run.
5. Comparison of multi objective function formulated for different tools highlighted the efficiency of using AlTiN coated tools. It had a gain of 47, 56 and 190% over uncoated, nACo coated, and TiSiN coated tools, respectively.
6. ANOVA of regression model also identified the tool coating parameter as the most effective with a contribution ratio of 41.64%. Speed and depth of cut were found to have contribution ratios of 18.82% and 8.10%, respectively.
7. Response surface optimization indicated optimum machining parameters of a speed of 15.36 m/min, feed of 1 µm/tooth, and a depth of cut of 71.81 µm with AlTiN coated tools. Confirmatory optimum experimental run resulted in reduced surface roughness and tool wear by 18% and 20%, respectively.

## 7. Future Recommendations

The outcome of the current study highlights certain future research endeavors. The significant increase in economy and productivity achieved during the course of this research can also be extended to other super alloys such as nickel and titanium alloys. In addition, machining can be carried out at high speeds for comparative analysis with low-speed machining. It is envisioned that the results obtained with the present study would go a long way in achieving sustainable development goals including those related to overall manufacturing system productivity.

## Figures and Tables

**Table 1:** Experimental setup.

Workpiece Material	Inconel 718
**Cutting length**	10 mm
**Flutes number**	2
**Diameter of tool**	0.5 mm
**Type of milling**	Full immersion

**Table 2:** Design of experiment.

Variables	Unit	Level 1	Level 2	Level 3	Level 4
**Coatings**	-	Un Coated	nACo	TiSiN	AlTiN
**Depth of cut**	µm	30	50	70	90
**Feed rate**	µm/tooth	0.25	0.50	0.75	1.00
**Cutting speed**	m/min	9	14	29	24

**Table 3:** Experimental design (Taguchi L16 array) with resulting burr formation, surface roughness, tool wear.

Run	Input Parameters				Output Parameters						
	Speed	Feed	DOC	Coating	Burr Height (µm)		Burr Width (µm)		Surface Roughness (µm)	Tool Wear (µm)	
	Vc (m/min)	fz (µm/tooth)	ap (µm)	(t_c)	Left	Right	Left	Right		Flute 1	Flute 2
1	9	0.25	30	nACo	106.502	266.057	344.247	495.801	0.072	95.556	79.532
2	9	0.5	50	AlTiN	152.281	455.755	308.902	479.949	0.083	41.507	39.867
3	9	0.75	70	TiSiN	245.546	215.663	491.352	394.318	0.098	65.724	54.562
4	9	1	90	Uncoated	85.934	495.0565	294.528	351.792	0.0985	30.745	41.008
5	14	0.25	50	TiSiN	53.672	449.7505	274.522	507.526	0.091	64.712	50.817
6	14	0.5	30	Uncoated	259.564	161.619	347.275	338.210	0.0535	40.082	33.858
7	14	0.75	90	nACo	129.014	192.0885	400.116	352.86	0.07	24.121	40.435
8	14	1	70	AlTiN	126.744	117.3685	384.435	240.660	0.0495	35.381	34.250
9	19	0.25	70	Uncoated	136.74	266.93	301.280	429.570	0.066	38.434	37.15
10	19	0.5	90	TiSiN	124.426	371.1955	390.436	382.47	0.0665	43.759	42.098
11	19	0.75	30	AlTiN	112.642	382.354	320.325	305.713	0.0495	43.405	50.236
12	19	1	50	nACo	89.494	89.247	415.859	250.424	0.0525	51.032	48.200
13	24	0.25	90	AlTiN	73.030	435.701	285.468	427.035	0.073	37.769	32.874
14	24	0.5	70	nACo	130.711	260.571	288.952	311.047	0.091	34.039	43.408
15	24	0.75	50	Uncoated	102.816	433.104	375.484	271.680	0.1095	38.453	39.307
16	24	1	30	TiSiN	256.522	413.469	416.327	502.212	0.1375	40.996	47.401

**Table 4:** Machining parameter combinations for individual best and worst responses.

Output Response														
	Best							Worst						
	Burr Height (µm)		Burr Width (µm)		Surface Roughness (µm)	Tool Wear (µm)		Burr Height (µm)		Burr Width (µm)		Surface Roughness (µm)	Tool Wear (µm)	
	Left	Right	Left	Right		Flute 1	Flute 2	Left	Right	Left	Right		Flute 1	Flute 2
Output results	49.972	94.715	251.716	209.042	0.0471	22.807	35.021	274.075	511.092	506.209	505.401	0.1411	98.572	90.017
Input response														
Speed Vc (m/min)	9	9	24	9	24	9	9	19	24	14	19	19	24	14
Feed fz (um/tooth)	0.5	0.75	0.25	0.25	1	0.25	0.25	0.25	0.25	1	0.75	0.5	1	0.5
DOC ap (um)	30	70	90	30	50	30	30	50	90	70	70	70	90	90
Coating (t_c)	TiSiN	TiSiN	TiSiN	TiSiN	TiSiN	TiSiN	nACo	nACo	AlTiN	nACo	Uncoated	AlTiN	Uncoated	Uncoated

**Table 5:** *GRG* values for burr formation, tool wear, and surface roughness; and *GRG* for each experiment.

Run	Input Parameters				Grey Relational Coefficient							Grey Relational Grade	Rank
	Speed	Feed	DOC	Coating	Burr Height (µm)		Burr Width (µm)		Surface Roughness (µm)	Tool Wear (µm)			
	Vc (m/min)	fz (µm/tooth)	ap (µm)	(t_c)	Left	Right	Left	Right		Flute 1	Flute 2		
1	9	0.25	30	nACo	0.6609	0.5344	0.6086	0.3434	0.6617	0.3333	0.3333	0.496504	14
2	9	0.5	50	AlTiN	0.5108	0.3563	0.7592	0.358	0.5677	0.6726	0.7694	0.570580	13
3	9	0.75	70	TiSiN	0.3492	0.6161	0.3333	0.4648	0.4757	0.4619	0.5182	0.459896	15
4	9	1	90	Uncoated	0.7614	0.3333	0.8442	0.5456	0.4731	0.8436	0.7415	0.648956	9
5	14	0.25	50	TiSiN	1	0.3601	1	0.3333	0.5146	0.4681	0.5653	0.605916	11
6	14	0.5	30	Uncoated	0.3333	0.7371	0.5984	0.5777	0.9167	0.6911	0.9595	0.687697	4
7	14	0.75	90	nACo	0.5774	0.6636	0.4633	0.5432	0.6822	1	0.7552	0.669283	5
8	14	1	70	AlTiN	0.5849	0.8783	0.4966	1	1	0.7603	0.9443	0.809189	1
9	19	0.25	70	Uncoated	0.5534	0.5331	0.802	0.4139	0.7273	0.7139	0.8451	0.655551	8
10	19	0.5	90	TiSiN	0.5927	0.4185	0.4833	0.4848	0.7213	0.6452	0.7167	0.580349	12
11	19	0.75	30	AlTiN	0.6358	0.4091	0.703	0.6723	1	0.6494	0.5733	0.663264	6
12	19	1	50	nACo	0.7419	1	0.4341	0.9318	0.9362	0.5703	0.6035	0.745394	2
13	24	0.25	90	AlTiN	0.8417	0.3693	0.9083	0.4172	0.6519	0.7235	1	0.701711	3
14	24	0.5	70	nACo	0.572	0.5422	0.8825	0.6547	0.5146	0.7827	0.6889	0.662513	7
15	24	0.75	50	Uncoated	0.6769	0.3711	0.5178	0.8114	0.4231	0.7136	0.7839	0.613963	10
16	24	1	30	TiSiN	0.3366	0.3849	0.4333	0.3378	0.3333	0.6791	0.6163	0.445915	16

**Table 6:** Analysis of variance for regression model.

Source	DF	Seq SS	Contribution	Adj SS	Adj MS	F-Value	*p*-Value
Model	13	0.142425	97.66%	0.142425	0.010956	6.41	0.143
Blocks	1	0.002137	1.47%	0.002462	0.002462	1.44	0.353
Linear	6	0.102811	70.50%	0.044197	0.007366	4.31	0.200
Speed	1	0.027451	18.82%	0.017566	0.017566	10.28	0.085
Feed	1	0.002815	1.93%	0.000091	0.000091	0.05	0.838
DoC	1	0.011811	8.10%	0.000485	0.000485	0.28	0.647
Coating	3	0.060734	41.64%	0.012982	0.004327	2.53	0.296
Square	3	0.027648	18.96%	0.020979	0.006993	4.09	0.203
Speed×Speed	1	0.021821	14.96%	0.015153	0.015153	8.87	0.097
Feed×Feed	1	0.002540	1.74%	0.002540	0.002540	1.49	0.347
DoC×DoC	1	0.003287	2.25%	0.003287	0.003287	1.92	0.300
2-Way interaction	3	0.009829	6.74%	0.009829	0.003276	1.92	0.361
Speed×Feed	1	0.004795	3.29%	0.004795	0.004795	2.81	0.236
Speed×DoC	1	0.003727	2.56%	0.003727	0.003727	2.18	0.278
Feed×DoC	1	0.001307	0.90%	0.001307	0.001307	0.77	0.474
Error	2	0.003416	2.34%	0.003416	0.001708		
Total	15	0.145841	100.00%				

**Table 7:** Comparison of optimized run with best initial experimental run.

	Input Parameters				Responses						
	Speed	Feed	DOC	Coating	Burr Height (µm)		Burr Width (µm)		Surface Roughness (µm)	Tool Wear (µm)	
	Vc (m/min)	fz (µm/Tooth)	ap (µm)	(t_c)	Left	Right	Left	Right		Flute 1	Flute 2
**Best run**	14	1	70	AlTiN	126.744	117.369	384.435	240.661	0.0495	35.381	34.251
**Optimized run**	15.3636	1	71.818	AlTiN	109.831	104.945	341.522	217.054	0.0407	28.618	27.104

## Data Availability

The data presented in this study are available upon request from the corresponding author.

## References

[1] Hazzan K.E., Pacella M., See T.L. (2021). Laser processing of hard and ultra-hard materials for micro-machining and surface engineering applications. Micromachines.

[2] Wojciechowski S. (2021). Estimation of Minimum Uncut Chip Thickness during Precision and Micro-Machining Processes of Various Materials—A Critical Review. Materials.

[3] M’Saoubi R., Axinte D., Soo S.L., Nobel C., Attia H., Kappmeyer G., Engin S., Sim W.M. (2015). High performance cutting of advanced aerospace alloys and composite materials. CIRP Ann.-Manuf. Technol..

[4] Chen N., Li H.N., Wu J., Li Z., Li L., Liu G., He N. (2021). Advances in micro milling: From tool fabrication to process outcomes. Int. J. Mach. Tools Manuf..

[5] Kladovasilakis N., Charalampous P., Tsongas K., Kostavelis I., Tzovaras D., Tzetzis D. (2022). Influence of Selective Laser Melting Additive Manufacturing Parameters in Inconel 718 Superalloy. Materials.

[6] Approaches A. (2021). Drilling Force Characterization during Inconel 718 Drilling: A Comparative Study between Numerical and. Materials.

[7] Bronis M., Miko E., Nowakowski L., Bartoszuk M. (2022). A Study of the Kinematics System in Drilling Inconel 718 for Improving of Hole Quality in the Aviation and Space Industries. Materials.

[8] Kiswanto G., Azmi M., Mandala A., Ko T.J. (2019). The Effect of Machining Parameters to the Surface Roughness in Low Speed Machining Micro-milling Inconel 718. IOP Conf. Ser. Mater. Sci. Eng..

[9] Schuster R., Kirchner V., Allongue P., Ertl G. (2000). Electrochemical micromachining. Science.

[10] Kock M., Kirchner V., Schuster R. (2003). Electrochemical micromachining with ultrashort voltage pulses-a versatile method with lithographical precision. Electrochim. Acta.

[11] Attanasio A. (2017). Tool run-out measurement in micro milling. Micromachines.

[12] Liu Y., Zhu D., Zhu L. (2012). Micro electrochemical milling of complex structures by using in situ fabricated cylindrical electrode. Int. J. Adv. Manuf. Technol..

[13] Xu L., Zhao C. (2017). Nanometer-scale accuracy electrochemical micromachining with adjustable inductance. Electrochim. Acta.

[14] Ling S., Li M., Liu Y., Wang K., Jiang Y. (2020). Improving machining localization and surface roughness in wire electrochemical micromachining using a rotating ultrasonic helix electrode. Micromachines.

[15] Allegri G., Colpani A., Ginestra P.S., Attanasio A. (2019). An experimental study on micro-milling of a medical grade Co-Cr-Mo alloy produced by selective laser melting. Materials.

[16] Wu M., Saxena K.K., Guo Z., Qian J., Reynaerts D. (2020). Fast fabrication of complex surficial micro-features using sequential lithography and jet electrochemical machining. Micromachines.

[17] Marrocco V., Modica F., Bellantone V., Medri V., Fassi I. (2020). Pulse-type influence on the micro-edm milling machinability of si3 n4–tin workpieces. Micromachines.

[18] Mian A.J., Driver N., Mativenga P.T. (2011). Identification of factors that dominate size effect in micro-machining. Int. J. Mach. Tools Manuf..

[19] Bissacco G., Hansen H.N., de Chiffre L. (2006). Size effects on surface generation in micro milling of hardened tool steel. CIRP Ann.-Manuf. Technol..

[20] Markopoulos A.P., Karkalos N.E., Mia M., Pimenov D.Y., Gupta M.K., Hegab H., Khanna N., Balogun V.A., Sharma S. (2020). Sustainability assessment, investigations, and modelling of slot milling characteristics in eco-benign machining of hardened steel. Metals.

[21] Azhdari Tadavani S., Shoja Razavi R., Vafaei R. (2017). Pulsed laser-assisted machining of Inconel 718 superalloy. Opt. Laser Technol..

[22] D’Addona D.M., Raykar S.J., Narke M.M. (2017). High Speed Machining of Inconel 718: Tool Wear and Surface Roughness Analysis. Procedia CIRP.

[23] Ucun I., Aslantas K., Bedir F. (2013). An experimental investigation of the effect of coating material on tool wear in micro milling of Inconel 718 super alloy. Wear.

[24] Bandhu D., Kumari S., Prajapati V., Saxena K.K., Abhishek K. (2020). Experimental investigation and optimization of RMDTM welding parameters for ASTM A387 grade 11 steel. Mater. Manuf. Process..

[25] Bandhu D., Abhishek K. (2021). Assessment of weld bead geometry in modified shortcircuiting gas metal arc welding process for low alloy steel. Mater. Manuf. Process..

[26] Chatterjee S., Mahapatra S.S., Behera A. (2022). NiTi joining with other metallic materials. Nickel-Titanium Smart Hybrid Mater.

[27] Lu X., Jia Z., Wang H., Si L., Wang X. (2016). Surface roughness prediction model of micro-milling Inconel 718 with consideration of tool wear. Int. J. Nanomanuf..

[28] Aslantas K., Hopa H.E., Percin M., Ucun I., Çicek A. (2016). Cutting performance of nano-crystalline diamond (NCD) coating in micro-milling of Ti6Al4V alloy. Precis. Eng..

[29] Özel T., Thepsonthi T., Ulutan D., Kaftanolu B. (2011). Experiments and finite element simulations on micro-milling of Ti-6Al-4V alloy with uncoated and cBN coated micro-tools. CIRP Ann.-Manuf. Technol..

[30] Aramcharoen A., Mativenga P.T., Yang S., Cooke K.E., Teer D.G. (2008). Evaluation and selection of hard coatings for micro milling of hardened tool steel. Int. J. Mach. Tools Manuf..

[31] Devillez A., Le Coz G., Dominiak S., Dudzinski D. (2011). Dry machining of Inconel 718, workpiece surface integrity. J. Mater. Process. Technol..

[32] Tansel I.N., Arkan T.T., Bao W.Y., Mahendrakar N., Shisler B., Smith D., McCool M. (2000). Tool wear estimation in micro-machining. Int. J. Mach. Tools Manuf..

[33] Attanasio A., Gelfi M., Pola A., Ceretti E., Giardini C. (2013). Influence of material microstructures in micromilling of Ti6Al4V alloy. Materials.

[34] Sun Z., To S. (2018). Effect of machining parameters and toolwear on surface uniformity in micro-milling. Micromachines.

[35] Aurich J.C., Bohley M., Reichenbach I.G., Kirsch B. (2017). Surface quality in micro milling: Influences of spindle and cutting parameters. CIRP Ann.-Manuf. Technol..

[36] Tayade P.M., Sorte P. (2021). Multi Objective Optimization in Micro Milling: Literature Review. https://www.researchgate.net/profile/Madhukar-B-Sorte/publication/352169609_Multi_Objective_Optimization_in_Micro_Milling_Literature_Review/links/60bcd890299bf10dff9d8a29/Multi-Objective-Optimization-in-Micro-Milling-Literature-Review.pdf.

[37] Joshi M., Ghadai R.K., Madhu S., Kalita K., Gao X.Z. (2021). Comparison of NSGA-II, MOALO and MODA for Multi-Objective Optimization of Micro-Machining Processes. Materials.

[38] Tien D.H., Duc Q.T., Van T.N., Nguyen N.T., Do Duc T., Duy T.N. (2021). Online monitoring and multi-objective optimisation of technological parameters in high-speed milling process. Int. J. Adv. Manuf. Technol..

[39] Kumar R., Bilga P.S., Singh S. (2017). Multi objective optimization using different methods of assigning weights to energy consumption responses, surface roughness and material removal rate during rough turning operation. J. Clean. Prod..

[40] Warsi S.S., Agha M.H., Ahmad R., Jaffery S.H.I., Khan M. (2019). Sustainable turning using multi-objective optimization: A study of Al 6061 T6 at high cutting speeds. Int. J. Adv. Manuf. Technol..

[41] Hughes J.I., Sharman A.R.C., Ridgway K. (2006). The effect of cutting tool material and edge geometry on tool life and workpiece surface integrity. Proc. Inst. Mech. Eng. Part B J. Eng. Manuf..

[42] Barry J., Byrne G., Lennon D. (2001). Observations on chip formation and acoustic emission in machining Ti-6Al-4V alloy. Int. J. Mach. Tools Manuf..

[43] Liu C.R., Mittal S. (1996). Single-step superfinish hard machining: Feasibility and feasible cutting conditions. Robot. Comput. Integr. Manuf..

[44] Zhou M., Chen Y., Zhang G. (2020). Force prediction and cutting-parameter optimization in micro-milling Al7075-T6 based on response surface method. Micromachines.

[45] Karna S.K. (2016). An Overview on Taguchi Method. Int. J. Eng. Math. Sci..

[46] Alghamdi S.S., John S., Choudhury N.R., Dutta N.K. (2021). Additive manufacturing of polymer materials: Progress, promise and challenges. Polymers.

[47] Khan M.A., Jaffery S.H.I., Khan M., Younas M., Butt S.I., Ahmad R., Warsi S.S. (2020). Multi-objective optimization of turning titanium-based alloy Ti-6Al-4V under dry, wet, and cryogenic conditions using gray relational analysis (GRA). Int. J. Adv. Manuf. Technol..

[48] Khan M.A., Jaffery S.H.I., Khan M., Younas M., Butt S.I., Ahmad R., Warsi S.S. (2019). Statistical analysis of energy consumption, tool wear and surface roughness in machining of Titanium alloy (Ti-6Al-4V) under dry, wet and cryogenic conditions. Mech. Sci..

[49] Komanduri R., Chandrasekaran N., Raff L.M. (1999). Some aspects of machining with negative-rake tools simulating grinding: A molecular dynamics simulation approach. Philos. Mag. B Phys. Condens. Matter Stat. Mech. Electron. Opt. Magn. Prop..

[50] Lu X., Jia Z., Wang H., Hu X., Li G., Si L. (2017). Measurement-based modelling of cutting forces in micro-milling of Inconel 718. Int. J. Nanomanuf..

[51] Platt T., Meijer A., Biermann D. (2020). Conduction-based thermally assisted micromilling process for cutting difficult-to-machine materials. J. Manuf. Mater. Process..

[52] Jaffery S.H.I., Khan M., Ali L., Mativenga P.T. (2016). Statistical analysis of process parameters in micromachining of Ti-6Al-4V alloy. Proc. Inst. Mech. Eng. Part B J. Eng. Manuf..

[53] Solheid J.S., Elkaseer A., Wunsch T., Scholz S., Seifert H.J., Pfleging W. (2022). Multiobjective Optimization of Laser Polishing of Additively Manufactured Ti-6Al-4V Parts for Minimum Surface Roughness and Heat-Affected Zone. Materials.

[54] Singh H., Patrange P., Saxena P. (2022). Multi-Objective Optimization of the Process Parameters in Electric Discharge Machining of 316L Porous Stainless Steel Using Metaheuristic Techniques. Materials.

[55] Algorithm A.B.C. (2022). Multiobjective Optimization of Heat-Treated Copper Tool Electrode on EMM Process Using Artificial Bee Colony (ABC) Algorithm. Materials.

[56] Chen H., Lu C., Liu Z., Shen C., Sun M. (2022). Multi-Response Optimisation of Automotive Door Using Grey Relational Analysis with Entropy Weights. Materials.

[57] Tan X., Deng J., Chen X. Generalized grey relational grade and grey relational order test. Proceedings of the IEEE International Conference on Systems, Man and Cybernetics.

[58] Bademlioglu A.H., Canbolat A.S., Kaynakli O. (2020). Multi-objective optimization of parameters affecting Organic Rankine Cycle performance characteristics with Taguchi-Grey Relational Analysis. Renew. Sustain. Energy Rev..

[59] Ziegel E.R. (1997). Taguchi Techniques for Quality Engineering. Technometrics.

